# Rare case of strangulated primary acquired perineal hernia causing small bowel obstruction requiring emergency operative repair

**DOI:** 10.1093/jscr/rjae164

**Published:** 2024-03-17

**Authors:** Giuleta Jamsari, Charlotte Kwik, Jenny King, Nimalan Pathma-Nathan

**Affiliations:** Department of Surgery, Westmead Hospital, Corner of Hawkesbury and Darcy Roads, Westmead, NSW 2145, Australia; Department of Colorectal Surgery, Westmead Hospital, Corner of Hawkesbury Road and Darcy Roads, Westmead, NSW 2145, Australia; Department of Obstetrics and Gynaecology, Westmead Hospital, Corner of Hawkesbury and Darcy Roads, Westmead, NSW 2145, Australia; Department of Colorectal Surgery, Westmead Hospital, Corner of Hawkesbury Road and Darcy Roads, Westmead, NSW 2145, Australia

**Keywords:** small bowel obstruction, perineal hernia, strangulation, laparotomy

## Abstract

Primary acquired perineal hernia is rare with only 100 reported cases in the literature. Emergency presentations of intestinal obstruction secondary to perineal hernia are very rare and to-date, there are only eight cases reported in the literature. We present a case of a 74-year-old lady who presented with a small bowel obstruction secondary to strangulated perineal hernia in the absence of pelvic exenteration or abdominoperineal resection requiring operative repair via combined open transabdominal and transperineal approach. To our knowledge, this case represents the first reported case of intestinal obstruction secondary to primary acquired perineal hernia in the absence of pelvic exenteration or abdominoperineal resection.

## Introduction

Perineal hernia, defined as the prolapse of intraperitoneal or extraperitoneal content secondary to a defect in the pelvic diaphragm, is uncommon with the majority of cases reported post pelvic exenteration or abdominoperineal resection (APR) [1]. Complication secondary to perineal herniae such as obstruction and strangulation are very rare with only eight reported cases in literature [2–9]. Due to its rare nature, clinical diagnosis and management are often challenging.

Herein, we present a rare case of perineal hernia in the absence of pelvic exenteration or APR leading to intestinal obstruction and strangulation requiring operative repair.

## Case report

A 74-year-old lady presented to our institution with acute onset colicky abdominal pain associated with abdominal distension and vomiting. She had previously undergone right nephrectomy for renal cell carcinoma, as well as an open total abdominal hysterectomy and bilateral salpingo-oophorectomy in 1990 for endometrial cancer with internal brachytherapy. A subsequent incisional hernia had been repaired with ‘Ultrapro’ mesh. She has had two previous instrumented vaginal delivery with forceps for prolonged labour and breech but had no previous history of colonic or rectal surgery. She also reported history of cystocele repair that was complicated by bladder perforation necessitating long-term indwelling catheterization, which has recurred following attempted surgical correction. Other relevant medical history includes hypertension, insulin-dependent type 2 diabetes, and dyslipidaemia.

**Figure 1:**
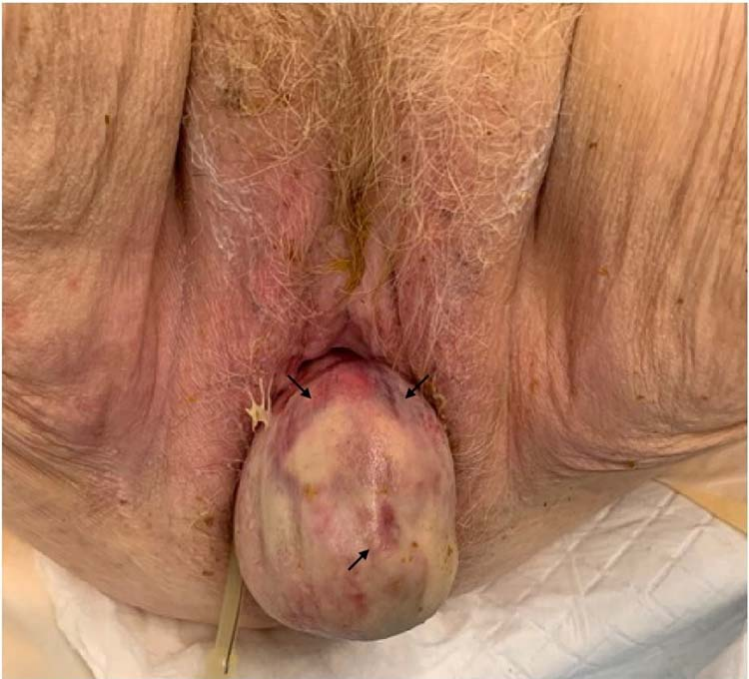
Small bowel prolapsing through vaginal vault associated with mottling of mucosa (indicated by the arrow).

On physical examination, she was haemodynamically stable and afebrile. Her abdomen was distended and generally tender without peritonism. Vaginal examination revealed a large mass prolapsing through the vagina, the mucosa of which was mottled, boggy, and tender to touch (Fig. 1). There were no clinical signs of peritonitis present.

Biochemistry showed an elevated white cell count to 12.7 × 10^9^/L (3.9–11.1 × 10^9^/L) with neutrophils to 10.1 × 10^9^/L (2.0–8.0 × 10^9^/L) and C-reactive protein of 367 mg/L (<4 mg/L). She also had acute kidney injury with creatinine of 220 μmol/L (45–90 μmol/L).

**Figure 2:**
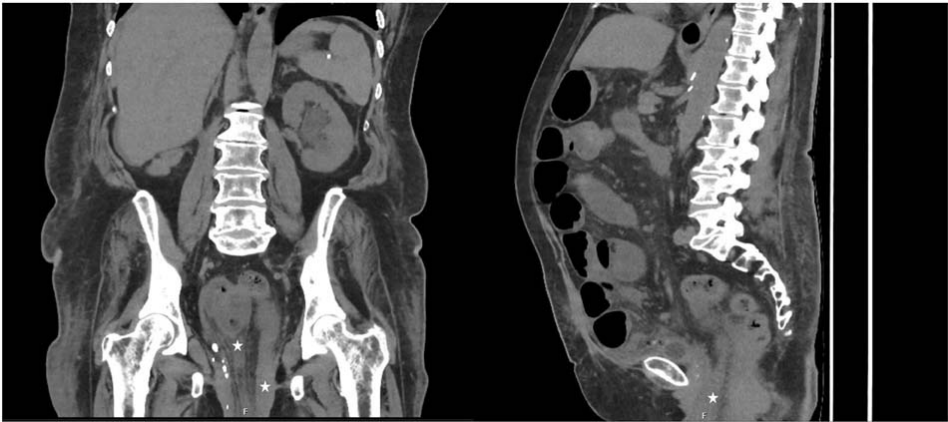
CT coronal (left) and sagittal (right) of abdomen and pelvis, demonstrating extension of small bowel into the pelvis (marked with star) consistent with perineal hernia.

Computed tomography (CT) of the abdomen and pelvis demonstrated evidence of small bowel obstruction and perineal hernia a 5 cm defect containing a loop dilated small bowel with mesenteric congestion. There was collapsed small bowel exiting the hernia defect. Overall, the clinical and radiological findings were suggestive of incarcerated small bowel that had herniated through a perineal hernia and prolapsed through the vaginal vault (Fig. 2).

**Figure 3:**
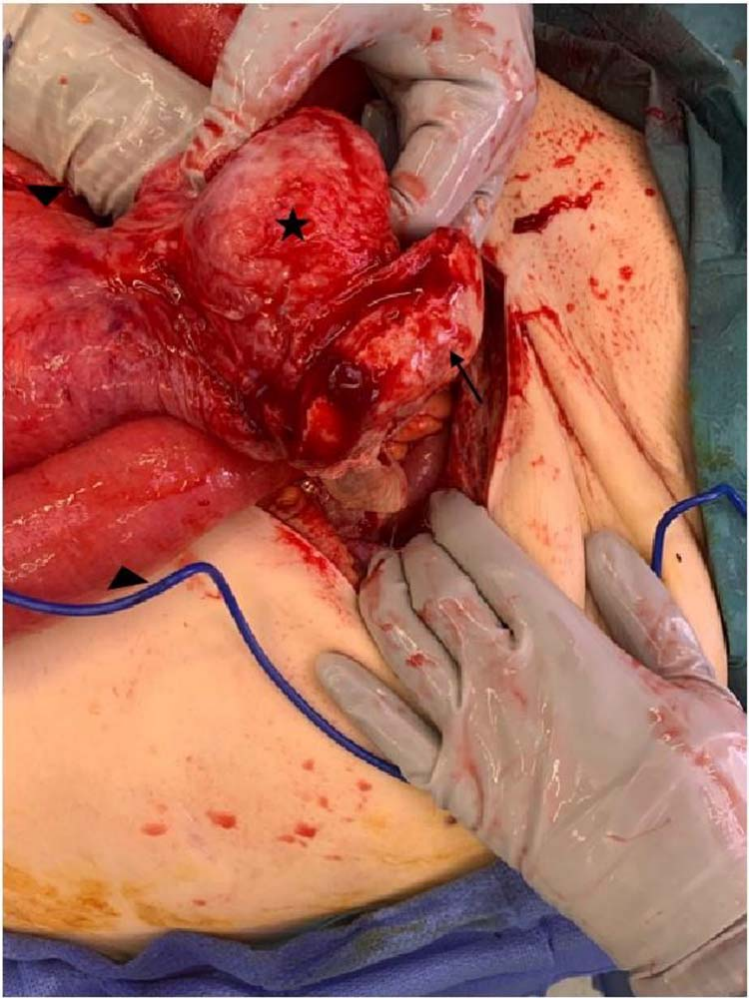
Intraoperative image demonstrating loop of small bowel (marked by triangle) extending into the perineal hernia sac (marked by star) with the vaginal vault mucosa attached to sac (marked by arrow).

The patient was admitted, decompressed with a nasogastric tube, and resuscitated prior to being booked for urgent laparotomy due to concern for possible strangulated bowel in context of mesenteric congestion and high inflammatory markers. Intraoperatively, a loop of small bowel contained within a chronic hernia sac was found to have herniated through a large defect in the pelvic floor and prolapsed through the vaginal vault (Fig. 3). The incarcerated small bowel was delivered using a combination of reduction from perineum and retraction from the abdominal cavity through a midline laparotomy incision. Approximately 20 cm of small bowel was resected and a primary stapled anastomosis was performed. Following delivery of herniated small bowel, the defect in the vaginal vault was stapled off and a well vascularized omental flap was placed in the pelvis. A transperineal repair of vaginal prolapse was subsequently performed for which the posterior vaginal epithelium opened to the defect and sacrospinous fixation was performed with Capio device. A transvaginal supris mesh was then secured along the vault line.

She made good post-operative recovery with return of gut function 72 h post-surgery and discharged from hospital on Day 10 post-surgery. At 12 months postoperatively, she has normal bowel function and no recurrence of perineal hernia.

## Discussion

Perineal hernia refers to protrusion of intra-abdominal viscera through a weakened pelvic floor and can be categorized aetiologically into primary and secondary. Primary perineal hernia is divided into congenital and acquired with acquired primary perineal hernia being uncommon with only 100 reported cases [8]. The predisposing factors include female gender, increase in intra-abdominal pressure such as in pregnancy and childbirth, obesity, smoking, history of hysterectomy, and previous pelvic chemoradiotherapy [10]. Secondary perineal hernia is more common, typically occurs following surgical procedure and commonly associated with pelvic exenteration or APR for pelvic colorectal surgery.

The diagnosis of perineal hernia in a patient without previous history of pelvic surgery is difficult and majority of patients with perineal hernia are asymptomatic. Clinical manifestations include perineal pain when sitting or standing upright, protruding gluteal or perineal lump, urinary dysfunction, and intestinal obstruction [10]. Intestinal obstruction associated with strangulation is rare and to our knowledge, this is the only case report of intestinal obstruction with strangulation in a patient with primary acquired perineal hernia requiring emergency repair.

As there are only limited cases of intestinal obstruction secondary to strangulated perineal hernia, there is no standardized approach to surgical management. Transabdominal (open or laparoscopic), transperineal, and combined abdominoperineal approach has been described in the literature with a systematic review showing combination approach having the lowest recurrence rate in repair of secondary perineal hernia [10]. Majority of the reported cases described open transabdominal approach due to presence of obstruction and compromised bowel [3, 5, 6, 8, 9], two cases reported laparoscopic repair of the strangulated perineal hernia [2, 4]. Reconstruction with tissue or mesh were used in the three of the cases with majority opted for biological mesh due to lower risk of infection [3, 4, 7]. For this case, due to the acute nature of the presentation with compromised small bowel, a combined open transabdominal and transperineal approach was performed with omentum placed within the pelvis. Mesh was not used intraabdominally due to the risk of infection associated with compromised bowel requiring resection.

## Conclusion

This is a rare case of perineal hernia in the absence of pelvic colorectal surgery, highlighting the importance in recognizing that perineal hernias can cause intestinal obstruction and strangulation. It also serves as reminder to consider perineal hernia as an underlying cause to intestinal obstruction in a patient with multiple predisposing factors as described in this case.
